# Supplementary material for: The causal relationship between the human gut microbiota and pyogenic arthritis: a Mendelian randomization study
Source: Front Cell Infect Microbiol. 2024 Nov 26;14:1452480. doi: 10.3389/fcimb.2024.1452480 (PMC11629706; doi:10.3389/fcimb.2024.1452480)
Supplement: Supplementary file 1 [file DataSheet1.zip › mendelian test/ebi-a-GCST90027669.csv_forest.pdf]

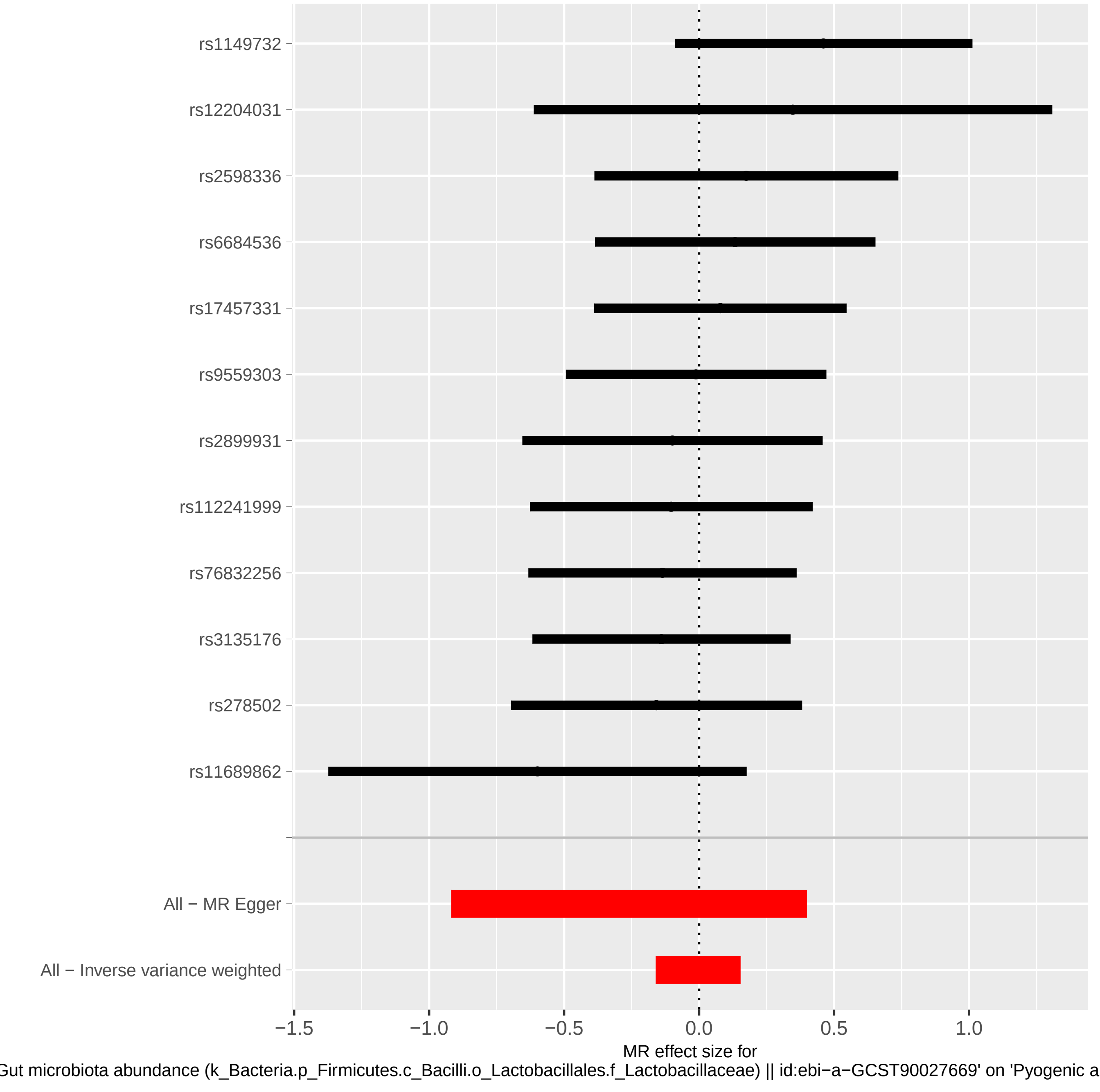

rs1149732
rs12204031
rs2598336
rs6684536
rs17457331
rs9559303
rs2899931
rs112241999
rs76832256
rs3135176
rs278502
rs11689862
All − MR Egger
All − Inverse variance weighted
−1.5
−1.0
−0.5
0.0
0.5
1.0
MR effect size for
Gut microbiota abundance (k_Bacteria.p_Firmicutes.c_Bacilli.o_Lactobacillales.f_Lactobacillaceae) || id:ebi−a−GCST90027669' on 'Pyogenic a
